# Multimodal Interdisciplinary Management of Level III Renal Cell Carcinoma With Inferior Vena Cava Tumor Thrombus: The Role of Preoperative Embolization, Intracaval Balloon Control, and Radical Nephrectomy With Inferior Vena Cava Thrombectomy

**DOI:** 10.7759/cureus.110930

**Published:** 2026-06-15

**Authors:** Faris Abushamma, Ibraheem Alami, Mohammed Tawfiq Abu Aisheh, Khaled Demyati, Abdelkarim Barqawi, Maha Akkawi, Mohammad Khader, Razan Odeh, Daher Alsadi, Moutaz W Sweileh, Ahmed Ghanem

**Affiliations:** 1 Department of Medicine, Faculty of Medicine and Allied Medical Sciences, An-Najah National University, Nablus, PSE; 2 Department of Urology, An-Najah National University Hospital, Nablus, PSE; 3 Department of General Surgery, An-Najah National University Hospital, Nablus, PSE; 4 Department of Pathology, An-Najah National University Hospital, Nablus, PSE; 5 Department of Radiology, An-Najah National University Hospital, Nablus, PSE; 6 Department of Hemato-oncology, An-Najah National University Hospital, Nablus, PSE

**Keywords:** endovascular balloon control, inferior vena cava tumor thrombus, intraoperative cavography, ivc thrombectomy, level iii, multidisciplinary, preoperative renal artery embolization, renal cell carcinoma

## Abstract

Renal cell carcinoma with tumor thrombus extending into the inferior vena cava (IVC) above the hepatic veins (Mayo level III) is among the most technically demanding scenarios in urooncological surgery. We describe the case of a previously healthy 59-year-old gentleman who presented with a large right renal mass and a 9 cm tumor thrombus extending from the right renal vein into the intrahepatic IVC. After multidisciplinary discussion, the patient underwent a combined urovascular procedure preceded by a same-day interventional radiology (IR) intervention comprising selective right renal artery embolization, embolization of the highly vascular caval tumor thrombus, and the placement of an accommodating intracaval balloon for suprahepatic vascular control. A rooftop incision was used for radical nephrectomy with IVC thrombectomy under triple-level caval control and contralateral renal vein control; intraoperative cavography by the IR team confirmed complete extraction of the thrombus and a patent IVC. Estimated blood loss was minimal, and no transfusion was required. The combination of preoperative selective embolization, intracaval balloon assistance, and intraoperative cavography may improve hemostatic control, reduce the risk of intraoperative tumor embolization, and provide a reproducible adjunct to conventional surgical thrombectomy for level III IVC tumor thrombi. Further prospective studies and larger case series are mandated to define the incremental benefit of each component.

## Introduction

Renal cell carcinoma (RCC) accounts for approximately 2-3% of all adult malignancies and is one of the most common urological cancers worldwide [1]. One of its distinctive biological characteristics is the propensity to invade the venous system: macroscopic extension of tumor thrombus into the renal vein and/or the inferior vena cava (IVC) is encountered in 4-10% of patients with newly diagnosed disease [2,3].

The Mayo classification stratifies caval tumor thrombi into four levels according to their cephalad extent. Level III thrombi extend above the hepatic veins but remain below the diaphragm, constituting the surgical inflection point at which conventional infrahepatic vascular control is no longer sufficient. The European Association of Urology 2025 guidelines, in concert with major contemporary series, consider radical nephrectomy with en bloc IVC thrombectomy as the gold standard, offering the only realistic prospect for long-term cure in nonmetastatic disease [1,4]. However, the operation is associated with substantial morbidity, major Clavien-Dindo complication rates as high as 38%, and a perioperative mortality of 4-10%, mainly driven by massive hemorrhage, intraoperative tumor fragmentation and pulmonary embolism, hepatic and renal dysfunction, and prolonged operative times [5,6].

In an attempt to mitigate these risks, several centers have integrated interventional radiology (IR) techniques into the perioperative pathway. Preoperative renal artery embolization has been shown to reduce intraoperative bleeding and transfusion requirements in nephrectomy for locally advanced disease and IVC thrombus [7,8]. More recently, endovascular balloon occlusion of the suprahepatic IVC, and even endovascular "downstaging" of level III and IV thrombi, have been proposed as alternatives to cardiopulmonary bypass and as adjuncts to conventional thrombectomy [9-11].

Despite these emerging tools, integrated multimodal interdisciplinary pathways that combine same-day IR embolization of both the renal artery and the caval thrombus, intracaval balloon assistance, conventional open surgical exposure, and intraoperative cavography remain scarcely described. They are not yet incorporated into routine practice in many regions. We therefore report a case of level III RCC IVC tumor thrombus managed through such an integrated pathway, with a focus on the conceptual and practical value of the intracaval balloon and of intraoperative cavographic confirmation of thrombus clearance.

## Case presentation

A previously healthy 59-year-old gentleman presented in January 2026 with a four-week history of right flank pain. He had no hematuria, no constitutional symptoms, and no significant past medical, surgical, or family history. Physical examination was unremarkable. Routine laboratory studies were within normal limits, with a baseline serum creatinine of 1.18 mg/dL and an estimated glomerular filtration rate of 71 mL/min/1.73 m². The remainder of his hematological and biochemical profile, including liver function tests and coagulation parameters, was unremarkable. Initial contrast-enhanced abdominopelvic computed tomography (CT) (Figure 1) demonstrated a heterogeneously enhancing right renal mass measuring approximately 5 × 6 cm, with extension into the right renal vein and IVC. Staging CT of the chest, abdomen, and pelvis then confirmed the renal lesion and the venous extension and excluded pulmonary metastatic disease. Dedicated abdominal magnetic resonance imaging (MRI) (Figure 2), which is the imaging modality of choice for delineating the cephalad extent of caval tumor thrombus [1], confirmed an exophytic, heterogeneously enhancing right renal mass measuring 9 × 7 × 8.5 cm (right-left × anteroposterior × craniocaudal), with extension into the perinephric fat. Tumor thrombus was seen extending from the right renal vein into the IVC and reaching its intrahepatic segment, with a maximal craniocaudal length of 9 cm, corresponding to a Mayo level III thrombus reaching just at the level of hepatic veins. In addition, a well-defined soft-tissue nodule (2.7 × 1.9 cm) was identified within the peritoneal cavity, slightly enlarged compared with the prior CT, of unknown etiology. Following multidisciplinary team (MDT) discussion, the recommendation was to obtain a biopsy of the small lesion and proceed with radical nephrectomy and thrombectomy if there was no evidence of metastasis. Biopsy showed a low-grade mesenchymal spindle-cell neoplasm.

**Figure 1:**
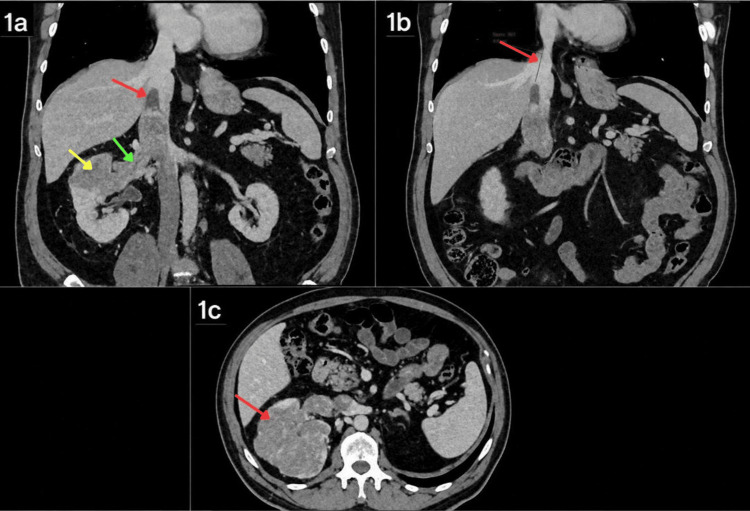
Contrast-enhanced CT images of the abdomen (1a) Coronal CT scan in the venous phase demonstrating a right RCC (yellow arrow) with tumor thrombus extending through the right renal vein to the IVC (green arrow), reaching just at the level of the hepatic veins (red arrow). (1b) Coronal CT scan indicates that the distance between the proximal thrombus and the cavoatrial junction measures 4 cm. (1c) Axial CT scan in the corticomedullary phase reveals a 10.5 x 9.8 cm solid mass in the upper and mid poles of the right kidney. CT: computed tomography, RCC: renal cell carcinoma, IVC: inferior vena cava

**Figure 2:**
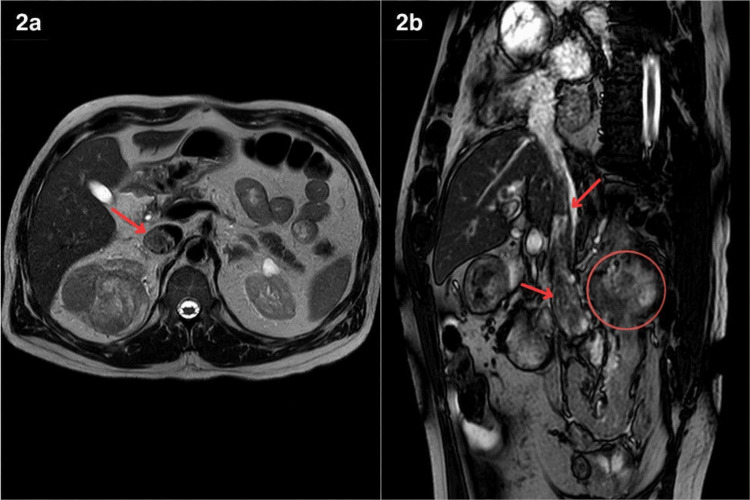
MRI of the abdomen demonstrating the right renal lesion (2a) Axial T2WI at the level of the right kidney showing the thrombus in the IVC. (2b) Sagittal MRI (BTFE) showing the right kidney with RCC (red circle) and the tumor thrombus extending to the IVC (red arrows). MRI: magnetic resonance imaging, T2WI: T2-weighted imaging, IVC: inferior vena cava, BTFE: balanced turbo field echo, RCC: renal cell carcinoma

Interdisciplinary planning and preoperative IR intervention

The case was reviewed in a dedicated MDT meeting attended by urology, hepatobiliary and vascular surgery, medical oncology, IR, cardiothoracic surgery, and anesthesia and intensive care. After thorough informed consent, the MDT consensus was to proceed with expedited radical right nephrectomy and IVC thrombectomy under combined urovascular control, with explicit acknowledgment of the substantial risks of major hemorrhage, intraoperative pulmonary tumor embolism, injury to surrounding structures, perioperative morbidity, and a mortality risk that may approach 30% in complex level III cases [5,6]. On the morning of surgery, the patient was transferred to the IR suite under monitored anesthesia. Through right common femoral arterial access, a selective right renal angiogram was obtained, and the main right renal artery was embolized to angiographic stasis using Contour 250-350 microns, then 510-700 microns, then 710-1000 microns, followed by 3 pushable coils (Figure 3). Particular attention was paid to the highly vascular caval component of the tumor thrombus: super-selective microcatheterization of feeding vessels arising from the renal capsular and lumbar circulation enabled targeted embolization of the intracaval thrombus itself, with the aim of devascularizing the tumor bulk inside the IVC and reducing the risk of intraoperative hemorrhage from the cavotomy site. Through a right jugular venous access and under fluoroscopic guidance, a large-volume accommodating compliant occlusion balloon (Medtronic, Reliant compliant stent graft balloon catheter 46 mm x 100 cm) was then advanced into the IVC and positioned cephalad to the proximal extent of the thrombus in the suprahepatic IVC just below the cavoatrial junction. The balloon was test-inflated to confirm wall apposition and complete venous occlusion under cavographic control, then deflated and left in situ on a stiff guidewire for intraoperative use. The patient was then transferred immediately to the operating room. The proposed advantages of the intracaval balloon and the conceptual reason for its central place in our pathway are fourfold: (i) prevention of cephalad dislocation or fragmentation of tumor thrombus during surgical mobilization, with consequent mitigation of the risk of intraoperative pulmonary tumor embolism; (ii) improved hemostatic control of the suprahepatic IVC without the need for extensive hepatic mobilization, division of the short hepatic veins, or thoraco-abdominal extension of the incision; (iii) availability of immediate intraluminal suprahepatic vascular control that can be redeployed instantaneously if a cephalad shift of the thrombus or a brisk venous bleed is encountered intraoperatively; and (iv) in selected cases, the possibility of “endovascular downstaging”-controlled caudal reduction or repositioning of the thrombus by endovascular manipulation may shorten the intracaval working segment and, in carefully selected anatomy, effectively convert a level III thrombus into a more manageable level II thrombus, as recently proposed in the literature [9,10].

**Figure 3:**
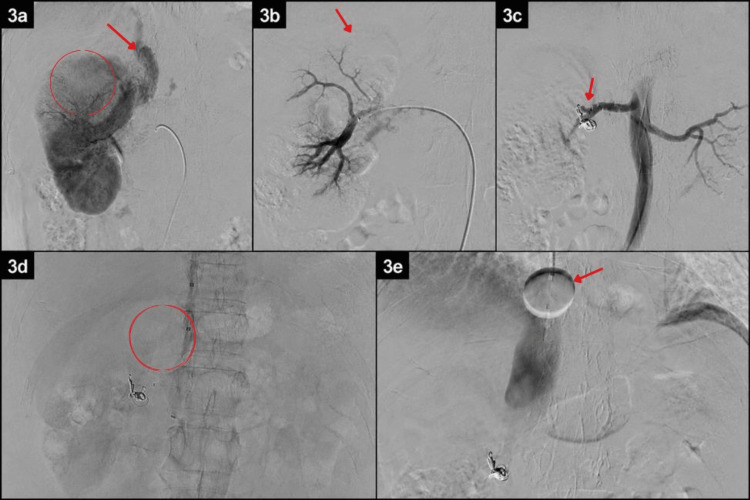
Angiographic and embolization procedure images of the right renal lesion (3a) Selective renal angiogram demonstrating a hypervascular right renal mass (red circle) and tumoral thrombus extending to the IVC (red arrow), with an intense, heterogeneous tumor blush fed by hypertrophied intra-parenchymal branches. (3b) Post-embolization angiogram shows complete devascularization and an absence of the previously noted tumor and thrombus blush. (3c) Post right renal artery occlusion with absence of renal parenchymal vascularity and tumor blush. (3d) Venacavogram from the right jugular vein through the balloon catheter, showing a filling defect representing the tumor-thrombus within the IVC. (3e) Venacavogram from the right jugular vein through the balloon catheter, while the balloon is inflated and with complete occlusion of the IVC, without reflux to the hepatic veins. IVC: inferior vena cava

Surgical technique

A rooftop incision was used to provide wide simultaneous access to the right renal fossa and to the entire infradiaphragmatic IVC. The procedure was performed jointly by the urological and the hepatobiliary surgical teams. After division of the falciform and right triangular ligaments and limited mobilization of the right lobe of the liver, the IVC was carefully skeletonized from the bifurcation up to its suprahepatic segment. Sequential vascular control was obtained using the vessel loops and snuggers (Rummel) technique at three levels of the IVC: infrarenal, suprarenal/infrahepatic, and suprahepatic, in addition to controlling the left renal vein. The intracaval balloon previously deployed by the IR team provided additional intra-luminal suprahepatic control during this dissection and obviated the need for extensive bare-area mobilization of the liver. A standard right radical nephrectomy was completed, with early ligation of the already embolized right renal artery, and the kidney was prepared for en bloc delivery with the thrombus. The IVC was then incised longitudinally along the renal ostium, and the tumor thrombus was extracted en bloc with the specimen after vascular control had been established at three levels: infrarenal, contralateral renal vein, and subhepatic after pushing the thrombus down, as it is a mobile thrombus as detected by hand feeling and intraoperative US (Figure 4). The cavotomy was closed primarily with a continuous 5-0 polypropylene suture using standard back-bleeding maneuvers to evacuate residual debris. The contralateral renal vein and IVC were sequentially de-aired, and the snares were released in a controlled, pre-defined sequence (infrahepatic, then suprahepatic, then left renal vein, then infrarenal). The total closure time of the IVC was 12 minutes. Once surgical hemostasis was secured, the IR team performed an intraoperative cavogram through the previously placed femoral venous sheath. The cavogram demonstrated a patent IVC with smooth contours, no residual intraluminal filling defect, no extravasation at the cavotomy site, and normal flow into the right atrium. Intraoperative cavographic confirmation of complete thrombus clearance is conceptually attractive in this setting because it provides real-time, intraluminal evidence of the completeness of resection at a moment when further surgical revision is still feasible, and it may complement intraoperative ultrasound and transesophageal echocardiography in delineating disjointed lumina, residual mural disease, and collateral pathways [12]. After confirmation of clearance and patency, the intracaval balloon was deflated and removed, the jugular sheath was withdrawn with manual compression, and the rooftop incision was closed in layers over closed-suction abdominal drains. The small left peritoneal lesion was excised completely. Total operative time was four hours; estimated intraoperative blood loss was minimal, and no intraoperative blood transfusion was required.

**Figure 4:**
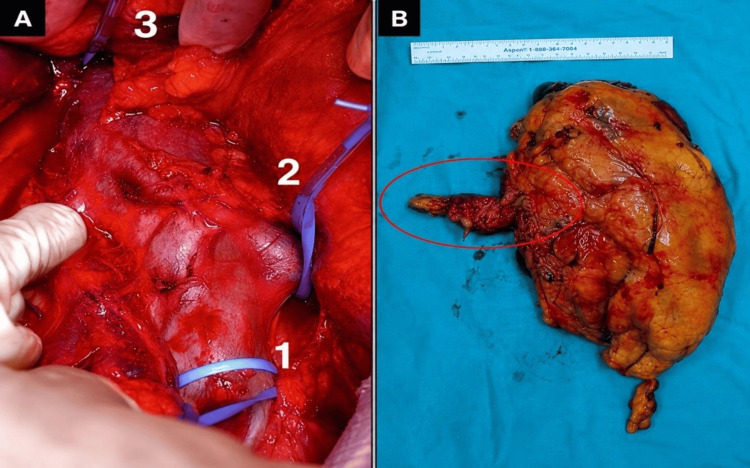
Intraoperative vascular control and extracted nephrectomy specimen with level III IVC tumor thrombus (4A) Intraoperative photograph demonstrating circumferential vascular control of the IVC prior to cavotomy and tumor thrombectomy. Three vessel loops are visible, encircling: (1) the infrarenal IVC, (2) the contralateral (left) renal vein, and (3) the infrahepatic IVC. An additional suprahepatic IVC control loop was placed above the hepatic veins to permit total hepatic vascular exclusion if required; this loop lies outside the field of view and is not visible in the photograph. This four-point isolation strategy enabled near-complete interruption of venous inflow and outflow within the affected IVC segment, providing optimal hemostatic control and exposure during cavotomy and tumor thrombus extraction. (4B) Corresponding en bloc radical nephrectomy specimen following complete excision, with the tumor thrombus (encircled) extending from the renal vein in a characteristic sausage-like configuration, illustrating the macroscopic extent of intraluminal venous involvement. IVC: inferior vena cava

Postoperative course

The patient was extubated in the operating theater immediately following completion of the procedure and was transferred to the surgical intensive care unit (ICU) for routine postoperative monitoring. He remained hemodynamically stable throughout the early postoperative period and did not require inotropic or vasopressor support. Postoperative hemoglobin showed only a mild decrease, consistent with the minimal intraoperative blood loss; no blood transfusion was administered, the abdominal drain output remained serous throughout, and the early postoperative serum creatinine was 1.6 mg/dL, consistent with the expected functional impact of the removal of a single, partially functioning kidney. ICU length of stay was two days, and total hospital length of stay was four days. No major Clavien-Dindo grade III-V complications were recorded; minor events included postoperative ileus during the first two postoperative days, which resolved with adequate mobilization and physiotherapy. The patient was discharged in a stable condition on appropriate venous thromboembolism prophylaxis, with arrangements for early outpatient oncological follow-up. Definitive histopathology of the nephrectomy specimen revealed clear cell RCC, WHO grade 3, with absence of perinephric fat invasion, absence of microscopic invasion of the IVC wall, and necrotic tumor thrombus present at the vascular margin; no definite viable tumor identified. The small left-sided lesion was consistent with a low-grade sarcoma. At the most recent follow-up one week postoperatively, the patient remained clinically well, with no significant complaints, and demonstrated normal functional status and performance.

## Discussion

Caval tumor thrombus is one of the most challenging complications of locally advanced RCC. Approximately 4-10% of patients with RCC present with macroscopic venous extension, and roughly one-third of these have a level III or IV thrombus that mandates complex vascular control [2,3]. Despite advances in surgical technique, perioperative imaging, and multidisciplinary care, contemporary single-center series of Mayo III-IV thrombectomy continue to report major complication rates of approximately 30-40%, perioperative mortality of 4-10%, median operative times in the range of 300-400 minutes, and median blood loss of 1.5-2.5 L [5,6,13]. Five-year overall and disease-free survival following complete resection are typically reported to be in the range of 50-65%, with the extent of thrombus, perinephric fat invasion, IVC wall invasion, high Fuhrman grade, and nodal status all identified as independent adverse prognostic factors [6,13,14]. In this context, any technical adjunct that can reduce intraoperative bleeding, control the proximal IVC without thoraco-abdominal extension or cardiopulmonary bypass, and confirm completeness of resection is clinically attractive.

Conventional surgical thrombectomy for level III disease classically relies on extensive hepatic mobilization, including a Pringle maneuver, division of the short hepatic veins, and a piggyback liver-mobilization technique to obtain suprahepatic IVC control, with venovenous bypass or cardiopulmonary bypass reserved for retrohepatic or supradiaphragmatic disease [4,15]. These maneuvers add significantly to operative time, blood loss, and the risk of hepatic and renal dysfunction. The approach reported here differs in that the major vascular control burden is deliberately shared with the IR team through three explicit adjuncts: preoperative embolization of the renal arteries and intracaval thrombus, an accommodating intracaval balloon for suprahepatic occlusion, and intraoperative cavography.

Preoperative renal artery embolization prior to nephrectomy for locally advanced disease and IVC thrombus has been most rigorously evaluated in the UroCCR-48 (REINBOL) study and in subsequent single-center series, which have shown that immediate preoperative embolization is well tolerated, reduces intraoperative bleeding and transfusion requirements, and does not increase the rate or severity of postoperative complications [7,8]. Extension of the embolization strategy to the highly vascular caval component of the thrombus is conceptually attractive: devascularization of the intracaval tumor should both reduce bleeding from the cavotomy site and decrease the friability of the thrombus during mobilization, although high-quality comparative evidence remains limited. Intraoperative embolization of small caval feeding vessels has been described in selected hands and appears safe [12].

An additional noteworthy observation in our case was the pathological finding of a necrotic tumor thrombus with no definite viable tumor identified at the vascular margin. Given that targeted super-selective embolization of the feeding vessels supplying the intracaval component of the thrombus was performed immediately before surgery, it is plausible that embolization contributed to thrombus devascularization and subsequent necrosis. Although a direct causal relationship cannot be established from a single case and no dedicated pathological assessment of treatment-related changes was undertaken, this finding raises the possibility that selective embolization of caval feeding vessels may provide benefits beyond hemostatic control by inducing ischemic changes within the tumor thrombus itself. Further studies are required to determine whether this effect is reproducible and clinically meaningful.

The intracaval balloon strategy used in this case extends earlier experience with suprahepatic IVC balloon occlusion as an alternative to surgical clamping in difficult anatomy [16,17]. Several groups have reported the successful use of compliant occlusion balloons, pulmonary artery occlusion devices, and, more recently, dedicated resuscitative endovascular balloon devices to provide proximal control during open and robotic IVC thrombectomy, with the additional advantages of intraluminal stabilization of the thrombus and a low-profile working field [10,16,18]. A more ambitious extension of this concept is "endovascular downstaging," in which percutaneous mechanical downstaging of thrombus is used to push the cephalad portion of a level III or IV thrombus before open surgery, thereby converting a high-risk thrombectomy into a lower-risk procedure and potentially eliminating the need for cardiopulmonary bypass [9,11]. Our use of the balloon is consistent with the more conservative and mechanical end of this spectrum. Rather than aspirating the thrombus, the balloon was used to occlude the suprahepatic IVC, to prevent cephalad embolization, and, where appropriate, to allow controlled displacement of the thrombus to a level that is amenable to standard infra-hepatic control.

Intraoperative cavography is a third, comparatively underused element of our pathway. In our hands, it provided immediate intraluminal confirmation of complete thrombus extraction, of a smooth and patent cavotomy closure, and of unobstructed flow into the right atrium. Several authors have recommended cavography as a complement to transesophageal echocardiography and intraoperative ultrasound for assessing thrombus clearance, defining the degree of residual occlusion, and identifying anatomical variants that may compromise venous return [12,19]. In our view, its principal value lies in the ability to act on its findings while the patient is still on the operating table and the cavotomy is still accessible.

Several caveats deserve emphasis. First, this is a single case, and we cannot infer either superiority or non-inferiority over conventional approaches from a single observation; the favorable intraoperative hemodynamic profile, the minimal blood loss, and the absence of any transfusion requirement in this patient may reflect tumor biology, surgical experience, or chance as much as the adjuncts described here. Second, embolization, balloon placement, and intraoperative cavography add cost, logistical complexity, and a small but real risk of vascular access complications, contrast-induced nephropathy, and balloon migration. Third, the published literature on integrated IR-assisted IVC thrombectomy is dominated by small retrospective series and individual case reports, with substantial selection bias and limited comparative data [5,6,10,20]. The approach reported here should therefore be regarded as a promising adjunct rather than a definitive standard of care.

We believe that this case nevertheless illustrates two clinically useful principles. First, the routine integration of IR into the perioperative pathway for level III RCC tumor thrombi is feasible. It can be implemented in a tertiary academic center without dedicated on-site cardiothoracic and bypass infrastructure. Second, an MDT approach to such cases is recommended and may significantly mitigate the hemorrhagic, embolic, and oncological risks associated with complex IVC surgery. Further prospective, multi-institutional studies and larger case series are needed to formally quantify the incremental benefit of each adjunct and to identify the patient subgroups most likely to benefit.

## Conclusions

Multimodal interdisciplinary management of level III RCC with IVC tumor thrombus, combining preoperative renal artery and intracaval thrombus embolization, an accommodating intracaval balloon for suprahepatic occlusion, conventional open radical nephrectomy with triple-level IVC and contralateral renal vein control, and intraoperative cavographic confirmation of clearance, is feasible, was performed safely in this patient, and was associated, in our patient, with minimal blood loss, no requirement for transfusion, and an uncomplicated early postoperative course. This integrated pathway may improve hemostatic control, reduce the risk of intraoperative tumor embolization, and provide objective confirmation of complete thrombus extraction; however, it should be regarded as a promising adjunct rather than a definitive standard of care. Further prospective studies and larger case series are mandated to formally define its incremental benefit.
